# Understanding the clinical spectrum of complicated *Plasmodium vivax *malaria: a systematic review on the contributions of the Brazilian literature

**DOI:** 10.1186/1475-2875-11-12

**Published:** 2012-01-09

**Authors:** Marcus VG Lacerda, Maria PG Mourão, Márcia AA Alexandre, André M Siqueira, Belisa ML Magalhães, Flor E Martinez-Espinosa, Franklin S Santana Filho, Patrícia Brasil, Ana MRS Ventura, Mauro S Tada, Vanja SCD Couto, Antônio R Silva, Rita SU Silva, Maria GC Alecrim

**Affiliations:** 1 Fundação de Medicina Tropical Dr. Heitor Vieira Dourado, Av. Pedro Teixeira, 25, 69040-000, Manaus Amazonas, Brazil; 2 Universidade do Estado do Amazonas, Av. Pedro Teixeira, 25, 69040-000, Manaus Amazonas, Brazil; 3 Universidade Nilton Lins, Av. Prof. Nilton Lins, 3259, 69058-040, Manaus Amazonas, Brazil; 4 Instituto Leônidas e Maria Deane, FIOCRUZ Amazônia, R. Terezina, 476, 69057-070, Manaus Amazonas, Brazil; 5 Fundação Oswaldo Cruz, Av. Brasil, 4365, 21040-360 Rio de Janeiro, Brazil; 6 Instituto Evandro Chagas, Rodovia BR-316 km 7 s/n, 67030-000 Ananindeua, Brazil; 7 Instituto de Pesquisas em Patologias Tropicais, R. da Beira, 7671, Rodovia BR-360 km 3, Porto Velho, Rondônia, Brazil; 8 Secretaria de Saúde do Amapá, Av. FAB, 69, 68.900-000 Macapá, Amapá, Brazil; 9 Universidade Federal do Maranhão, Praça Madre Deus 2, 65025-560, São Luiz Maranhão, Brazil; 10 Universidade Federal do Acre, Rodovia BR-364 km 4, 6637, 69915-900, Rio Branco Acre, Brazil

**Keywords:** Malaria, *Plasmodium vivax*, Brazil, Severity, Clinical aspects, Anaemia, Systematic review

## Abstract

The resurgence of the malaria eradication agenda and the increasing number of severe manifestation reports has contributed to a renewed interested in the *Plasmodium vivax *infection. It is the most geographically widespread parasite causing human malaria, with around 2.85 billion people living under risk of infection. The Brazilian Amazon region reports more than 50% of the malaria cases in Latin America and since 1990 there is a marked predominance of this species, responsible for 85% of cases in 2009. However, only a few complicated cases of *P. vivax *have been reported from this region. A systematic review of the Brazilian indexed and non-indexed literature on complicated cases of vivax malaria was performed including published articles, masters' dissertations, doctoral theses and national congresses' abstracts. The following information was retrieved: patient characteristics (demographic, presence of co-morbidities and, whenever possible, associated genetic disorders); description of each major clinical manifestation. As a result, 27 articles, 28 abstracts from scientific events' annals and 13 theses/dissertations were found, only after 1987. Most of the reported information was described in small case series and case reports of patients from all the Amazonian states, and also in travellers from Brazilian non-endemic areas. The more relevant clinical complications were anaemia, thrombocytopaenia, jaundice and acute respiratory distress syndrome, present in all age groups, in addition to other more rare clinical pictures. Complications in pregnant women were also reported. Acute and chronic co-morbidities were frequent, however death was occasional. Clinical atypical cases of malaria are more frequent than published in the indexed literature, probably due to a publication bias. In the Brazilian Amazon (considered to be a low to moderate intensity area of transmission), clinical data are in accordance with the recent findings of severity described in diverse *P. vivax *endemic areas (especially anaemia in Southeast Asia), however in this region both children and adults are affected. Finally, gaps of knowledge and areas for future research are opportunely pointed out.

## Background

*Plasmodium vivax *is the most geographically widespread species of *Plasmodium *causing human disease, with most cases reported in Central and Southeast Asia, in the horn of Africa and in Latin America [1]. It is considered to be a potential cause of morbidity and mortality amongst the 2.85 billion people living at risk of infection, excluding the large African populations who are mostly Duffy negative and, therefore, naturally less susceptible to this infection. However recent data suggest that the parasite is evolving and may use alternative receptors other than Duffy (DARC) for erythrocyte invasion [2]. It is estimated that 5.5% of the population under risk live in the Americas [3]. The major biological characteristic of this parasite is the presence of liver hypnozoites responsible for the frequent relapses, which add a substantial number of cases to the general burden of the disease, what is being faced as one of the most challenging bottlenecks for vivax malaria eradication [4].

Although often regarded as causing a benign infection, there is recent increasing evidence that the overall burden, economic impact, and severity of *P. vivax *have been underestimated, in part due to a bias in the scientific literature which traditionally devoted most of its attention to the more lethal parasite *Plasmodium falciparum*, probably as a reflection of a more substantial funding [5]. Until 16 October 2011, the search in MEDLINE using *P. vivax *as keyword retrieved 5,026 indexed abstracts; using *P. falciparum*, on the other hand, retrieved almost five times more abstracts: 25,807. Even in places where *P. vivax *represents the major local problem to be tackled, clinical research is still focused on *P. falciparum *[6]. There is robust evidence in the past decade from hospital-based studies in India and Indonesia that *P. vivax *is able to cause severe disease [7,8]. Some authors argue that this clinical severity may only now be properly recognized and announced by researchers in the field, but these complications apparently are not new from a historical perspective [9]. Actually, the case fatality rate (CFR) related to malarial infections in the English marshes during the 16th and 17th centuries, corresponding to the Little Ice Age, suggest that *P. vivax *(a parasite more prone to persist in vectors even under low temperatures) may have killed part of this population already victimized by famine [10]. During the first half of the 20th century, malariotherapy in patients with neurosyphilis, using essentially the 'non-severe' *P. vivax *parasite, led to diverse complications, CFR ranging from 3.3 to 30.3% [11]. The major related complications in these co-infected patients were liver damage, ruptured spleen, jaundice, delirium, uncontrolled vomiting and persistent headaches [11]. That reinforces the concept that *P. vivax *infection may synergize with other co-morbidities resulting in more complicated disease. Added to local geographical and social determinants, wide Annual Parasite Incidence (API) and CFR variations due to this species are seen around the world.

In summary, *P. vivax*, which has long been neglected and mistakenly considered 'benign' [12], is receiving an increasing amount of importance in the debates taking place on malaria epidemiology and control, drug resistance, pathogenesis and vaccines [13]. As reviewed elsewhere, the good clinical characterization of severe disease in vivax infection is the first step to understand how the inflammatory response to this parasite contributes to pathogenesis [14].

Traditionally, Brazil has been responsible for almost half of all cases of malaria in Latin America. In 2009, 308,498 cases of malaria were reported in this country (257,571 caused by *P. vivax*), representing 54.9% of all the malaria reported in the Americas [15]. Cases are virtually restricted to the Amazon Basin (constituted by the states of Amazonas, Acre, Roraima, Amapá, Pará, Tocantins, Rondônia, and parts of Mato Grosso and Maranhão). Amazonian urban agglomerations under continuous economical development trigger intense migration flows, such as in the city of Manaus (in the Western Brazilian Amazon), helping to maintain the disease under endemic levels [16,17]. Malaria in Brazil is mostly related to *P. vivax *since the 1990s, when the available tools for control at the moment were put together and intensified, such as the fast diagnosis through thick blood smear (TBS) in all febrile patients, and free access to anti-malarials, integrated through a decentralized primary care-centred public health system [18]. Allied to that, an active community of local malariologists has been persistently identifying the profile of anti-malarial resistance with permanent counseling to the Brazilian Ministry of Health, which responds promptly to these evidences, changing the first line regimens [19]. As the sexual forms (which are infective for the vector) of *P. falciparum *generally appear later in the course of infection, opportune diagnosis and treatment tend to have a high impact on reducing the transmission intensity of this species but the same is not true for *P. vivax*, whose gametocytes are present in the very first days of the infection, before efficacious treatment is usually started. In 2008, 59% of all malaria cases registered in the Brazilian Amazon were treated in the first 48 h after appearance of symptoms (SIVEP-Malaria, 2009). These public health measures allied to a regularly updated online information system also impacted the number of deaths related to *P. falciparum*, which were not more than 58 in 2009 (Brazilian Ministry of Health, 2010). As a consequence, even in the non-indexed literature, severity due to *P. falciparum *is not frequently reported anymore in Brazil.

Brazil has reported 85% of its cases related to *P. vivax *in 2010, which puts this country in a peculiar epidemiological situation, as one of the few countries around the world with *P. vivax *predominance. The impact of *P. vivax*/*P. falciparum *co-infections or simultaneous circulation of both species with similar frequencies in a given population, upon the immunological status and clinical presentation of malaria is still unclear [20,21], but most probably clinical data from population from certain areas should not be extrapolated to other areas in distinct epidemiological conditions. Actually, the lack of data on clinical presentation of *P. vivax *infection allied to the several particularities of this region, including the diverse genetic background of its population, implicate that the generalization of the findings from Southeast Asia may be inappropriate.

In Brazil, in 1903, the young physician Carlos Chagas (most known for the discovery of American trypanosomiasis afterwards) wrote his MD thesis on the haematological complications of malaria, which, at that moment, also occurred in the non-Amazon area. His major findings in studying *P. vivax *patients were severe anaemia, splenomegaly, leukopenia, cachexia and jaundice associated to concomitant staphylococcal disease [22]. Bone marrows were also analysed in these patients with no conclusive findings. Later on, during the 1940s, Djalma Batista in Manaus described a series of malarial cases from his outpatient clinics in whom large splenomegaly, cachexia and minor bleeding were frequent among those with the 'benign' tertian malaria [23]. More recently, from 1998 to 2008, 234 deaths related to vivax disease were officially reported to the Brazilian Ministry of Health [18], and an increase in the hospitalization trends for vivax patients was published in a tertiary care hospital from Manaus [24]. To complicate matters, these facts parallel a lack of robust biomarkers and specific criteria for severe disease for this species in the literature. A *sine qua non *requisite in the analysis of clinical severity related to *P. vivax *infection is the exclusion of mixed infection with *P. falciparum *through a more sensitive technique such as PCR and the exclusion of other co-morbidities which may be responsible for the clinical presentation *per se*. In the literature, in general, reports of 'complicated/severe' cases lack more precise and uniform definition criteria, in part due to the rare application of more robust endpoints such as death and admission to the intensive care unit (ICU), and therefore end up suffering bias through individual judgment of authors, editors and reviewers. As in most of the data published there were no systematic exclusion of co-morbidities and/or mono-infection confirmation using PCR, performing a meta-analysis of severe manifestations of *P. vivax *becomes virtually impossible. The other bias in the case of Brazil is that many relevant data are confined in abstracts from national scientific meetings and graduate students' dissertations and theses. The systematic review of these unpublished data therefore could contribute to the understanding of the clinical spectrum of vivax infection in this country and ultimately as a representative sample from Latin American vivax malaria.

### Search strategy

The sources for published data on clinical aspects of vivax infection in Brazil were MEDLINE (1948 to February 2011) and LILACS (1982 to February 2011). The following search strategy was devised for both databases: (*Plasmodium vivax*).mp. AND (Brazil).mp. All types of study designs with primary data were included (cross-sectionals, case-controls, cohorts, case series and case reports). The abstracts were analysed in details by two independent reviewers and publications were selected if they mentioned any type of clinical complication (no specific criterion was used) in at least one patient with the diagnosis of vivax infection. Disagreement between the two reviewers was solved through consensus. Articles were excluded if they were reviews and also if they did not contain primary data on clinical aspects. For included studies, there were extracted data on date of publication, location, number of patients, and characteristics of participants (age range, pregnancy status, presence of co-morbidities), if molecular diagnosis through PCR was used to assess vivax malaria mono-infection and fatality. Exclusion criteria for analysis were participants with mixed infections (*P. falciparum/P. vivax*); studies in where patients with *P. falciparum *and *P. vivax *were both presented but the clinical data reported was not individualized for each species; and studies reporting the same patients from previous studies from the same authors. Through abstract analysis, 297 articles were retrieved and after application of the inclusion and exclusion criteria, 27 articles (from 1987 to 2011) were selected, which are presented in Table 1.

**Table 1:** Summary of publications retrieved from MEDLINE and LILACS referring to clinical aspects of *P. vivax *malarial patients in Brazil (1948-2011)

	Authors	Year of publication	Location City (State)	PCR confirmation	Age range (in years)	Number of patients	Type of clinical complication	Pregnancy	Death	Co-morbidity
1	Botelho et al. [25]	1987	Cuiabá (Mato Grosso)	No	NA	41	Pulmonary manifestations	No	No	NA

2	Moura et al. [26]	1987	NA	No	NA	1	Congenital malaria (?)	No	No	NA

3	Severo et al. [27]	1994	NA	No	NA	112	Hepatitis and jaundice	No	No	NA

4	Marques et al. [28]	1996	São Paulo (São Paulo)	No	5 weeks	1	Congenital malaria and anaemia	No	No	NA

5	Siqueira-Batista et al. [29]	1998	Rio de Janeiro (Rio de Janeiro)	No	62	1	Cardiac arrhythmia after chloroquine use	No	No	NA

6	Ventura et al. [30]	1999	Belém (Pará)	No	0-14	100	Anaemia	No	No	Hookworms and malnutrition

7	Pinheiro et al. [31]	2002	Belém (Pará)	No	NA	23	Low birth weight	Yes	No	NA

8	Jarude et al. [32]	2003	Rio Branco (Acre)	No	12-49	235	Anaemia, jaundice, hepatitis and hypoglycaemia	Yes	No	NA

9	Lacerda et al. [33]	2004	Manaus (Amazonas)	Yes	20	1	ITP	No	No	No

10	Silva et al. [34]	2004	Belém (Pará)	No	18-60	11	Blackwater fever after primaquine use	No	No	G6PD deficiency

11	Melo et al. [35]	2004	Manaus (Amazonas)	No	7-84	126	Ocular lesions	No	No	Hypovitaminosis A

12	Braga et al. [36]	2004	Fortaleza (Ceará)	No	25	1	Neurological symptoms	No	No	NA

13	Lomar et al. [37]	2005	São Paulo (São Paulo)	No	43	1	ARDS	No	No	No

14	Vermehren et al. [38]	2005	Manaus (Amazonas)	No	51	1	Acute renal failure	No	No	No

15	Braga et al. [39]	2006	Coari (Amazonas)	No	14-69	333	Hepatitis	No	No	HBV

16	Cabral et al. [40]	2006	Manaus (Amazonas)	No	4	1	Blackwater fever, anaemia and ARDS	No	No	Sickle cell anaemia

17	Lacerda et al. [41]	2007	Manaus (Amazonas)	No	23	1	Splenic hematoma	No	No	NA

18	Santana et al. [42]	2007	Manaus (Amazonas)	No	3-67	73	Methaemoglobinemia after primaquine use	No	No	G6PD deficiency

19	Lacerda et al. [43]	2008	Maués (Amazonas)	Yes	14	1	Chronic splenomegaly and thrombocytopaenia	No	No	NA

20	Ramos Jr et al. [44]	2010	Manaus (Amazonas)	No	8-39	18	Blackwater fever after primaquine use	No	No	G6PD deficiency

21	Alexandre et al. [45]	2010	Manaus (Amazonas)	Yes	0-80	17	Jaundice, anaemia, shock, acute renal failure, ARDS and haemoglobinuria	No	**Yes (1)**	HAV, hypertension and diabetes

22	Siqueira et al. [46]	2010	Manaus (Amazonas)	Yes	16	1	Rhabdomyolysis	No	No	No

23	Melo et al. [47]	2010	Careiro (Amazonas)	No	5-14	54	Anaemia	No	No	Intestinal helminthes

24	Andrade et al. [48]	2010	Buritis (Rondônia)	Yes	9-54	19	Jaundice, anaemia, shock, acute renal failure and ARDS	No	**Yes (6)**	No

25	Chagas et al. [49]	2010	Manaus (Amazonas)	No	9-44	411	Vaginal bleeding and amniorrexis	Yes	No	NA

26	Fragoso et al. [50]	2011	Manaus (Amazonas)	Yes	48	1	Hypovolaemic shock after CQ use	No	No	Hemophilia A

27	Ferreira et al. [51]	2011	Macapá (Amapá)	No	15-60	20	Methaemoglobinemia after primaquine use	No	No	G6PD deficiency

Unpublished studies were searched manually in the Annals of the Congress of the Brazilian Tropical Medicine Society (published in supplements of the indexed journal *Revista da Sociedade Brasileira de Medicina Tropical *[*Journal of the Brazilian Society of Tropical Medicine*]), from 1964 to 2011. This is the most traditional scientific event for tropical medicine clinicians in Brazil. Similar inclusion and exclusion criteria were used in this search. However if the same abstract data were published afterwards as a full paper, the published paper information was presented here. If the abstract referred to a dissertation or thesis, this more detailed information was presented instead. Forty-five abstracts were retrieved from 1995 to 2011. Of these, 17 fulfilled any of the exclusion criteria and therefore, 28 abstracts are presented in Table 2.

**Table 2:** Summary of abstracts referring to clinical aspects of *P. vivax *malarial patients published in the proceedings of the Annual Congress of the Brazilian Society of Tropical Medicine (1964-2011)

	Authors	Year of publication	Location City (State)	PCR confirmation	Age range (in years)	Number of patients	Type of clinical complication	Pregnancy	Death	Co-morbidity
1	Alecrim et al. [52]	1995	Manaus (Amazonas)	No	26	2	Splenic haematoma rupture	No	**Yes (1)**	NA

2	Kalmar et al. [53]	1998	São Paulo (São Paulo)	No	24	1	Pulmonary manifestations	No	No	NA

3	Sardinha et al. [54]	1998	Manaus (Amazonas)	No	42/57	2	Auto-immune anaemia induced by secondary cryoagglutinins	No	No	NA

4	Victoria et al. [55]	1998	Manaus (Amazonas)	No	21	1	ITP	No	No	NA

5	Zumpano et al. [56]	1998	Belo Horizonte (Minas Gerais)	No	40	1	Splenic haematoma rupture	No	No	NA

6	Silva et al. [57]	2000	Manaus (Amazonas)	No	NA	25	Thombocytopaenia and bleeding	No	No	NA

7	Aragão et al. [58]	2001	Manaus (Amazonas)	No	NA	50	Thombocytopaenia and bleeding	No	No	NA

8	Evangelista et al. [59]	2002	Manaus (Amazonas)	No	< 1 month	7	Congenital malaria (jaundice and anaemia)	No	No	NA

9	Moura et al. [60]	2002	Belo Horizonte (Minas Gerais)	No	69	1	Acute lung oedema	No	**Yes**	NA

10	Park et al. [61]	2002	São Paulo (São Paulo)	No	NA	237	Thrombocytopaenia	No	No	NA

11	Viana et al. [62]	2002	Santa Inês (Maranhão)	No	21	1	Pleural effusion	No	No	NA

12	Albuquerque et al. [63]	2003	Manaus (Amazonas)	No	NA	202	Anaemia, thrombocytopaenia, blackwater fever, jaundice and bleeding	No	No	Yes (diabetes, chronic renal failure, HIV, G6PD deficiency)

13	Lacerda et al. [64]	2003	Manaus (Amazonas)	Yes	46	1	Acute lung oedema	No	**Yes**	NA

14	Silva et al. [65]	2003	Belém (Pará)	No	21	1	Acute lung oedema	No	No	NA

15	Silva et al. [66]	2004	Tucuruí (Pará)	No	59	1	Vasculitis	No	**Yes**	NA

16	Tavares et al. [67]	2004	Manaus (Amazonas)	No	12	1	Splenic haematoma	No	No	NA

17	Penna et al. [68]	2005	Belo Horizonte (Minas Gerais)	No	52	1	Pulmonary manifestations	No	No	Yes (Dyslipidemia)

18	Mello et al. [69]	2006	Belém (Pará)	No	NA	1	Seizures and coma	No	No	NA

19	Menezes et al. [70]	2006	Manaus (Amazonas)	No	14-48	48	Abortion and premature delivery	Yes	No	NA

20	Oliveira et al. [71]	2006	Manaus (Amazonas)	No	22	1	Blackwater fever after primaquine use	No	**Yes**	Yes (G6PD deficiency)

21	Bastos et al. [72]	2007	Salvador (Bahia)	No	28	1	Jaundice and cholangitis	No	No	NA

22	Campos et al. [73]	2007	São Paulo (São Paulo)	No	NA	1	Splenic haematoma rupture	No	No	NA

23	Gurgel at al. [74]	2007	Manaus (Amazonas)	No	< 3 months	5	Congenital malaria (jaundice)	No	No	NA

24	Borzacov et al. [75]	2008	Porto Velho (Rondônia)	No	33	1	Acute psychosis after CQ use	No	No	NA

25	Cardoso et al. [76]	2008	Porto Velho (Rondônia)	No	57	1	Leukemoid reaction	No	No	NA

26	Ohnishi et al. [77]	2008	Belém (Pará)	No	29	1	Pulmonary manifestations	No	No	NA

27	Menezes et. al. [78]	2010	Manaus (Amazonas)	No	NA	421	Anaemia	Yes	No	NA

28	Ohnishi et al. [79]	2010	Belém (Pará)	No	> 15	83	Pulmonary manifestations and TNF and IL-12 polymorphisms	No	No	NA

Masters' dissertations and doctoral theses abstracts since 1987 were searched in the online database http://capesdw.capes.gov.br/capesdw/Teses.do maintained by the *Coordination for the Improvement of Higher Education Personnel *(CAPES), the institution which coordinates and supervises all the Brazilian Graduate Programmes in all areas of knowledge. The full original electronic documents were downloaded from the website when available or obtained through contact with the respective graduate students. Ten dissertations and three theses are presented in Table 3.

**Table 3:** Summary of master dissertations and doctoral theses retrieved from CAPES referring to clinical aspects of *P. vivax *malarial patients in Brazil (1948-2011)

	Authors	Year of publication	Location City (State)	PCR confirmation	Age range (in years)	Number of patients	Type of clinical complication	Pregnancy	Death	Co-morbidity
1	Urbaez-Brito JD [80]	1995	Costa Marques (Rondônia)	No	NA	35	Jaundice	No	No	HBV

2	Alecrim MGC [81]	2000	Manaus (Amazonas)	Yes	> 18	426	Thrombocytopaenia, bleeding and DIC	No	**Yes (1)**	NA

3	Neves, JJO [82]	2002	Belém (Pará)	No	NA	127	Thrombocytopaenia	No	No	NA

4	Marques HO [83]	2004	Manaus (Amazonas)	No	15-75	106	Thrombocytopaenia and coagulation disorders	No	No	NA

5	Oliveira MS [84]	2004	Manaus (Amazonas)	No	0-11	69	Anaemia	No	No	Intestinal helminthes

6	Silva IBA [85]	2004	Belém (Pará)	No	> 15	83	Thormbocytopaenia and TNF	No	No	NA

7	Raposo CCBS [86]	2006	Buriticupu (Maranhão)	No	1-70	140	Jaundice, anaemia, thrombocytopaenia, acute renal failure, ARDS, bleeding and neurological symptoms	No	No	No

8	Pereira MSS [87]	2006	Manaus (Amazonas)	No	NA	76	Jaundice	Yes	No	NA

9	Guerreiro NSV [88]	2006	Macapá (Amapá)	No	25-39	35	Anaemia	No	No	NA

10	Lacerda MVG [89]	2007	Manaus (Amazonas)	Yes	> 18	142	Thrombocytopaenia and bleeding	No	No	No

11	Silva SBR [90]	2009	Cuiabá (Mato Grosso)	No	NA	397	Thrombocytopaenia	No	No	NA

12	Fragoso SCP [91]	2010	Manaus (Amazonas)	Yes	1-88	17	Autopsies series (acute lung oedema, shock, blackwater fever, spleen haematoma rupture)	No	**Yes (17)**	Pneumonia, G6PD deficiency, chronic hepatitis, HIV and yellow fever

13	Lança EFC [92]	2011	Manaus (Amazonas)	No	0-14	24	Anaemia, shock, ARDS, coma, jaundice, blackwater fever, metabolic acidosis and hypoglycaemia	No	**Yes (2)**	Malnutrition, gastroenteritis, sepsis, G6PD deficiency

### Major clinical complications

Classical malaria paroxysms are typically short and sharply delineated within a period of less than eight hours. Fever is one feature that is almost invariably present during a paroxysm. Any of other common symptoms of the febrile syndrome, such as chills, rigours and sweating, are also described. These symptoms of a paroxysm could be accompanied by others, including headache, nausea and vomiting, and moderate to severe muscle, joint and back pain [93]. Indeed high fever tends to be more evident in vivax disease even with lower parasitaemia, due to its recognized lower fever-threshold (around 100 infected RBCs/microlitre) [94]. Therefore, any description of these classical symptoms, together or isolated, should be regarded by any experienced clinical as non-severe malaria, regardless of their intensity, because they are not associated to increased rates of hospitalization or fatality.

In the Brazilian literature reviewed, a wide spectrum of clinical complications aside from the classical symptoms of vivax malaria was found throughout the 68 indexed and non-indexed publications, despite the low number of deaths attributed to this species in this literature sample. The major complications are addressed as follows:

### Anaemia

World Health Organization (WHO) criterion for severe anaemia is haemoglobin below 5 g/dL in children and under 7 g/dL in adults. However the clinical manifestations due to anaemia *per se *are not known and to what extent it contributes to the respiratory distress associated with the hyperdynamic status of the febrile syndrome. There is scarce literature on malarial anaemia in population-based studies in Latin America, as reviewed elsewhere [95]. On top of that, major differences in Latin America are seen when the same methodology is applied. That is probably related to distinct genetic background and environmental factors, e.g. in the Amazon Basin (intense racial mixture) and in the Colombian Pacific Coast (non-mixed black population) [96]. It is not known if anaemia is as frequent among patients from Brazil as in Southeast Asian patients, where *P. vivax *is considered to be a disease of children because the acquisition of immunity against this species occurs much faster than for *P. falciparum*, in highly endemic areas [97]. In Brazil only 25% of vivax disease affects children 0-14 years of age, however severe anaemia was reported in hospitalized children and adults, needing red blood cell (RBC) transfusions [45]. A key description of anaemia in vivax malaria children in Latin America was published in Venezuela in 2006 [98]. The 'congenital malaria' in newborns from the present series of reports with severe anaemia confirms previous findings that vivax malaria has an important clinical impact in children under 3 months [99]. Non-severe anaemia, however, seems to be as frequent as 25.8% among the population of a recent occupation area in Rondônia, where hydroelectric power plants are being built [100]. The cut-off of haemoglobin under 12 g/dL as a criterion of anaemia however should be seen with scepticism because of age ranges and the lack of baseline levels of haemoglobin validated to specific populations, which makes meta-analyses susceptible to misclassification. Major confounding factors in the global analysis of anaemia are the local contributors to this haematological complication such as iron-deficiency anaemia, which was found to occur in 5.6% of a rural Amazonian population, mostly among school children and women [101]. Another important associated condition, which may interfere in the comparison between distinct populations, is the prevalence of intestinal helminthic infection. In a study performed with anaemic children, the presence of hookworms and malnutrition was cited [30]. However some controversy exists regarding this influence since in a cohort study, children with any intestinal helminth were protected from anaemia triggered by acute vivax infection [47]. In fact anti-helminthic treatment and iron supplementation reduced the haematological indexes in the population from an endemic area for malaria [102]. No Brazilian study has addressed the concomitant diagnosis of parvovirus B19 as a contributing factor to anaemia in malaria, considering that recent evidence supports that the use of chloroquine (CQ) may stimulate viral replication in the bone marrow, worsening anaemia [103]. Apparently pregnant women develop anaemia as a major complication in vivax infection [32,78], and the impact upon the concept needs further investigation. Chronic co-morbidities affecting erythrocyte physiology, such as sickle cell anaemia (SCA), may be related to more severe haemolysis and severe anaemia as well [40].

### Thrombocytopaenia and other coagulation disorders

Thrombocytopaenia as defined by platelet counts under 150,000/μL seems to be very frequent among patients with vivax malaria and apparently more frequent in vivax than in falciparum patients [104], despite not being a consensus [105]. The increase in the report of thrombocytopaenia in several reference centres could also be a reflection of a better laboratorial infrastructure. Only in recent decades in developing countries automated full blood counts included platelet count as a routine. Many studies in Brazil confirm that platelet counts are directly correlated to peripheral parasitaemia [89,90], but the meaning of this finding is still unknown. However, only mild bleeding is usually associated with this haematological complication in studies where detailed and systematic clinical description of the patients was made, even for severe thrombocytopaenia, which means in general platelet count under 50,000/μL [81,89,90]. In fact, there is no report in the whole literature of a fatal case of patient presenting exclusively with severe thrombocytopaenia, even for *P. falciparum*. That is probably why thrombocytopaenia, regardless of being described as a complication by WHO, is not strictly-speaking considered a severity criterion by itself [106]. What happens most of the time is that thrombocytopaenia is usually taken as a surrogate marker for DIC in settings where no specific examinations to confirm this severe complication are available, such as prothrombin activation time, D-dimers and fibrin degradation products. However, there is a disproportionate difference in the proportions of thrombocytopaenia, which is considered relatively frequent in large studies for frequency estimation [90] and of DIC, which is a rare complication, very scarcely reported in the literature associated to *P. vivax *infection [107,108]. Actually, there is some coagulation cascade activation, but usually with minor impact on coagulation tests and platelet counts [83]. It is important to consider however that in areas where dengue is also endemic, as is the case of Brazil, thrombocytopaenia studies should obligatorily rule out this viral infection, which also presents a substantial percentage of thrombocytopaenia as part of its non-severe presentation [109]. In fact there are cases of co-infection already reported in the Brazilian Amazon recently [110], but the literature poorly describes the clinical aspects of this coincidental infection [111].

### Respiratory distress and pulmonary oedema

Respiratory distress is defined by oxygen saturation less than 94%, or deep breathing (acidotic breathing), or an age-stratified increased rapid respiratory rate (> 32/min in adults, > 40 in children 5-14 y, > 50 in children aged 2 mo to 5 y, and > 60 in babies less than 2 mo)[112]. However this syndromic approach does not translate any mechanism of disease and may be associated to the clinical presentation of febrile syndrome during the malarial paroxysm, severe anaemia, metabolic acidosis, lung oedema, pneumonia or acute respiratory distress syndrome (ARDS). In most of the cited Brazilian studies, there are no described criteria on how respiratory distress was defined, which makes comparisons with the general literature impossible. Sometimes imprecise clinical presentation is simply defined as pulmonary manifestations. In only one, ARDS is well characterized, comprising detailed radiological characterization and arterial gas analysis (FiO_2_/PaO_2_) [37]. Lung oedema is usually based on clinical and radiological parameters and the effect of fluid overload is not clear for vivax infection, since only a few cases were reported so far, Brazilian cases included [39,64,65,113,114]. The impairment of respiratory symptoms after the beginning of treatment with CQ referred elsewhere [115] was not mentioned in any of the present reports, which could be due to inappropriate study design. Ruling out pneumonia is not easy because of the low frequency of positive blood cultures and due to the fact that in most of these patients with pulmonary complications empirical antibiotics are initiated as a rule. Data from the Papuan Indonesia indicate that many infants who die with *P. vivax *have radiological evidence of pneumonia [116], but the specificity of radiological findings to differentiate vivax-induced pulmonary abnormalities from pneumonia is questionable. In Mozambique, pyogenic bronchopneumonia was a common cause of respiratory distress in autopsied pregnant women with falciparum malaria, in both HIV positive and negative [117]. In the Amazon, HIV prevalence is estimated to be ~1% (unpublished data), which makes opportunistic diseases less prone to impact on severe clinical complications of vivax malaria, as is the case for falciparum malaria in Africa.

### Neurological syndrome

This is classically the most lethal clinical complication of severe falciparum malaria and the definition is also very imprecise with a wide spectrum of possible presentations, such as: impaired consciousness or unrousable coma (Glasgow coma score ≤10 or Blantyre coma scale ≤2); prostration, i.e. generalized weakness so that the patient is unable walk or sit up without assistance; failure to feed; or multiple convulsions (more than two episodes in 24 h). Despite being infrequent in our studies, the phenomenon was also reported but not only in children [36,86]. These reports must be very cautious in terms of ruling out other malarial complications as the cause of the neurological manifestations, such as hypoglycaemia and metabolic acidosis, but also associated infections as bacterial or viral meningoencephalitis. In India, acute intermittent porphyria was an unexpected co-morbidity associated to the neurological manifestations of patients with vivax malaria [118]. In Papua, *P. vivax*-associated coma was rare, occurring 23 times less frequently than that seen with falciparum malaria, and was associated with a high proportion of non-malarial causes and mixed infections detected using PCR [119].

### Acute renal failure

This complication is suspected in cases of oliguria and confirmed if serum creatinine is higher than 3.0 mg/dL. Bacterial sepsis, dehydration, shock and past history of chronic renal failure should be routinely searched in the differential diagnosis. It was also reported in the Brazilian literature [38,48], but in one study one case was found in a patient with arterial hypertension, what could be a triggering condition [45]. Despite not being frequent in Brazil, *Plasmodium malariae *is found in some scattered areas [120], and as a potential cause of glomerulonephritis [121], this parasite should be ruled out by molecular biology tools whenever acute renal failure is detected in a malarial patient with vivax infection, due to similarities of these two species at routine optical microscopy.

### Jaundice

New WHO guidelines already point to hyperbilirubinaemia (total bilirubin > 3.0 mg/dL) as being a weak marker of severity, unless it is followed by any other vital organ dysfunction [106]. This finding seems to be the most frequent among children and adults with vivax disease considered as 'severe' [122,123]. Since haemolysis is not usually as severe as to cause significant clinical jaundice, most of these patients actually have some hepatocyte necrosis as evidenced by the mild to moderate liver enzymes (AST/ALT) increase with subsequent cholestasis [124]. It was shown that icteric syndrome was a common cause of hospitalization in pregnant women with vivax malaria in Manaus [87]. It was also detected in newborns [59,74], which makes vivax malaria an obligatory differential diagnosis of neonatal sepsis. Jaundice in the presence of vomiting and upper abdominal pain should raise suspicion on acalculous cholecystitis, a poorly described complication apparently with good prognosis [125]. Other diseases that may evolve to an icteric syndrome may be ruled out, especially because they are also more frequent in the tropics, such as leptospirosis [34] and typhoid fever [126,127]. Hepatitis A virus (HAV) and vivax co-infection has already been reported as cause of jaundice and high elevation of transaminases [45]. Hepatitis B virus (HBV) is also highly prevalent in Brazil, especially in the Amazon [128] and there is some evidence that *P. vivax*/HBV co-infection may be related to more frequent jaundice [80] and higher transaminase levels.

### Algid malaria

Algid malaria refers to the shock syndrome usually defined as circulatory collapse (systolic pressure under 70 mmHg in adults or under 50 mmHg in children) non-responsive to fluids. In the present vivax malaria reports, it was more reported most frequently among patients who died, suggesting that, as expected for this severe clinical complication, it could be regarded as a good marker of severity. However, the aetiology of this complication is still unclear even for *P. falciparum*. Apparently it is multifactorial and the complication should be regarded as a syndrome where cardiac dysfunction, dehydration, bleeding, adrenal insufficiency, and bacterial sepsis could all play a role [129]. A review of all malaria deaths in the USA found that 5% were due to *P. vivax *associated with cardiac disease [130], which suggests cardiac dysfunction as a contributing factor to algid malaria. There is robust evidence that bacteraemia in Africa is associated with higher fatality in falciparum malaria in children [131]. Less frequently shock occurs isolated, but usually as part of multi-organ dysfunction syndrome (MODS), leading to a clinical picture suggestive of 'malaria-induced toxic shock' [132].

### Metabolic complications

Metabolic acidosis (plasma bicarbonate < 15 mmol/L) and hyperlactataemia (lactate > 5 mmol/L), which are common in severe falciparum malaria and are good predictors of fatal outcome, have never been described in vivax severe disease. In a series of children with vivax infection admitted to the ICU, metabolic acidosis is mentioned [92], however concomitant sepsis is described in this series and specificity for malaria cannot be assumed. If one admits lactic acidosis as a consequence of hypoxia triggered by microvasculature obstruction in falciparum disease, the scarcity of data on the frequency of this phenomenon in vivax disease may simply reflect the less severe obstruction due to less cytoadhesion, as already suggested elsewhere [133,134]. In the case of hypoglycaemia (blood glucose < 40 mg/dL), the complication has been rarely described elsewhere [123,135], and in only two studies in Brazil this finding was reported among children and pregnant women [32,92].

### Pregnancy-associated malaria

The impact of vivax infection upon pregnancy and the concept is less clear in Brazil and Latin America as a whole, despite robust evidence that vivax malaria causes low birth weight and maternal anaemia exists in Thailand [136] and Indonesia [137]. The burden of the infection due to this species in Brazilian pregnant women from a highly endemic area in the Amazon seems to be high [138]. Malaria anaemia in pregnant women with vivax is already known [137] and data from Brazil confirm that this is the most common complication among these women [78]. Additionally the few reports in the present series also point to low birth weight, vaginal bleeding, amniorrhexis, abortion, premature delivery, hypoglycaemia, hepatitis and jaundice as complications [31,32,49,70,87]. *Hyperemesis gravidarum *may superimpose to the febrile syndrome and to the gastrointestinal side effects associated to CQ in pregnant women, contributing to uncontrolled vomiting and consequent metabolic disorders. Apparently in the case of pregnancy, co-morbidities do not seem to be frequent among patients with clinical complications. Despite the need of more pathogenesis studies with the infected placenta, ultrasound studies in order to search for prognostic markers are urgently needed.

### Atypical complications

Some atypical complications are not frequently described for malaria and likewise are not classically referred as severe malaria. Rhabdomyolysis has been reported for vivax in 1993 in a patient with myoadenylate deaminase deficiency [139]; only one case was reported in Brazil in a patient without co-morbidities [46]. Rarely, patients with vivax malaria could evolve with immune thrombocytopenic purpura (ITP) as a complication of the acute infection [33]. To confirm this diagnosis, the patient has to be followed up with persistent thrombocytopaenia for many weeks after the efficacious anti-malarial treatment and diseases, in which ITP is more frequently seen, such as HIV, should be discarded. The mechanisms involved are poorly understood. Splenomegaly is considered a typical finding in the physical examination of a patient with vivax disease, but the occurrence of spleen haematomas evolving with rupture and fatal outcome is relatively rare [41,52,91] despite being more frequent among this species as compared to falciparum [140]. In any case, patients with vivax malaria referring abdominal pain should be investigated for this complication as some patients may evolve with a bad prognosis if not properly managed by a surgeon. Ocular manifestations in vivax disease apparently have no relation to cerebral malaria or bad prognosis, as is the case for falciparum [141]. Few reports have been published on vivax patients with non-severe disease and retinal haemorrhage [142] and in Brazil this fundoscopical finding was associated with hypovitaminosis A [35]. Another atypical complication, which may be more frequent than expected for vivax infection, and with outstanding impact upon the development of some emerging economies in the globe, is poor school performance which should be a surrogate marker for the intellectual impairment related to malaria [143]. Acute malnutrition has been shown to be a complication of vivax malaria in highly endemic areas [144]. In Brazil a few evidences show that malnourishment and vivax co-exist but the impact of this association is still unknown [145]. In only two studies was malnutrition referred to as a possible cause of the reported clinical complication [92,146]. Vasculitis [66], leukemoid reaction [76] and pleural effusion [62] as a marker of severity seems to be speculative and details of these reports do not support any in-depth analysis.

### Other aspects

High parasite density as a marker of severity for *P. vivax*, as it is for *P. falciparum*, still needs additional studies, considering this parasite infects preferably reticulocytes. The same occurs with the presence of schizonts in peripheral blood, which is usually associated with high sequestered biomass and severity for falciparum [147], but is still an unexplored aspect for vivax. Strong linear trends were identified regarding increasing plasma levels of C reactive protein (CRP) and the gradation of disease severity [48]. Super-oxide dismutase-1 (SOD-1) seems to be a powerful predictor of disease severity in individuals with different clinical presentations of vivax malaria [148].

As soon as precise markers of severity are available, it would be possible to design studies powered to analyse the influence of the host genetics in the development of severe vivax disease. Some association between pulmonary manifestations and TNF and IL-12 polymorphisms has been attempted [79]. It has been proposed for the first time in Manaus that G6PD deficiency could protect against vivax malaria, in a cross-sectional study, based on past history of the enrolled population [149]. This protection was later confirmed in Pakistan [150]. Male hemizygotes for this deficiency also showed to be protected against severe falciparum malaria [151]. No data exist on the protection against severe vivax disease. Likewise, people with the FYA/FYB genotype presented higher susceptibility to clinical vivax malaria [152]. Since the discovery in Brazil that Duffy-negative individuals could be infected by *P. vivax *[153], some speculation on the other possible invasion receptors has emerged. However, cohort studies are needed to investigate the real impact of the distinct Duffy genotypes on clinical malaria incidence, submicroscopic asymptomatic infection, malaria-triggered anaemia and lower parasitaemia, as already suggested for FYB/FYX and FYA/FYX genotypes in the Brazilian Amazon [154].

### Advances in pathogenic mechanisms

The major advance in the study of the pathogenesis of severe vivax disease was the demonstration of *P. vivax*-infected RBCs cythoadhesion on human lung endothelial cells (HLEC) and placental tissue *ex vivo *[134]. This cythoadhesion was obviously lower than *P. falciparum*-infected RBCs adhesion, but with similar stability. However the next challenge is to try to link this finding to the *in vivo *phenomena [155]. The increased adhesion with the addition of LPS in the *P. vivax ex vivo *model suggests that endothelial activation may be an enhancing event. The role of augmented platelet-derived microparticles [156] and CD4^+^CD25^+^FoxP3^+ ^regulatory T cells (Tregs) cells found in vivax disease should also be investigated in severe disease. Plasma levels of TNF, IFN-γ and also IFN-γ/IL-10 ratios were increased and exhibited a linear trend with gradual augmentation of disease severity [48]. Patients with severe disease also presented higher haemolysis and higher plasma concentrations of Cu/Zn SOD-1 and lower concentrations of PGE-2 and TGF-β than those with mild disease [157]. Oxidative stress was also proposed as a mechanism for thrombocytopaenia found in vivax disease [158,159], as well as its association with TNF [85]. Circulating immune complexes were not associated to vivax thrombocytopaenia [89], but polymorphisms of the highly immunogenic AMA-1 were associated to platelet count in these patients [160], suggesting that immunological mechanisms are involved in platelet destruction. In the case of anaemia, there is no correlation between the presence of anti-erythrocyte and anti-cardiolipin antibodies and the presence or intensity of this haematological finding [161]. Auto-immunity induced by secondary cryoagglutinins should be explored [54]. Erythropoiesis seems to be affected [162], and the finding of parasites inside the bone marrow [43] stimulate the search for mechanisms of diserythropoiesis in this milieu, despite technical limitations to analyse this tissue in humans. The role of the spleen in severe disease is still unknown, as well as the role of the variant subtelomeric multigene vir family, which may influence the sequestration of infected RBCs in this organ [163]. Parasite genetics, such as MSP-1 and CSP polymorphisms, has not been shown to be associated with clinical severity [164].

In summary, the immune response in patients with severe vivax disease has not been fully addressed in the general literature, and further approaches are needed in order to unveil immune mechanisms related to these complications.

### Vivax malaria therapy concerns

In terms of therapy, CQ and primaquine (PQ) are still the drugs of choice for the treatment of vivax malaria in many endemic areas, Brazil included. It is important however to keep in mind that side effects of these drugs could be erroneously taken as clinical severity associated to the parasite infection. In the case of CQ, it is considered a safe drug, despite the occurrence of pruritus, which most of the time is considered to be a minor effect and rarely requires the drug withdrawal [165]. Psychosis on the other hand is a more severe complication [75,166], as well as cardiac arrhythmia [29]. Atypical complications of its use such as severe gastric bleeding were associated with haemophilia A [50]. In the case of PQ, tranquillity is not the same as with CQ, because PQ is able to induce metahaemoglobinaemia [42,51] and severe haemolysis [44] in patients with G6PD deficiency. The burden of the deficiency in Brazil is poorly measured but the few data available in endemic areas for malaria has shown it to be between 3.0% [149] and 5.8% [167] among men, since the deficiency is linked to the X-chromosome. In the case of Brazil, the prescription of PQ in the abbreviated regimen (0.5 mg/kg/day for 7 days) without any routine G6PD screening may contribute to increase the frequency and severity of the side effects triggered by this drug, as confirmed by the reports of patients with blackwater fever after PQ use, including one fatal case [63,71,91,92,168]. To complicate matters, for the radical cure, the new drug under late stage clinical investigation, tafenoquine, shows no evidence that it is safer than PQ in G6PD deficient [169].

The simultaneous occurrence of severe vivax disease and CQ-resistance in some countries has raised the question of a possible association between severity and resistance, especially for anaemia [170]. CQ resistance actually has been reported in Brazil almost at the same time as clinical severity [171,172], but some studies argue against that, showing that severe patients responded to CQ [45]. Added to that, reliable genetic markers of resistance are lacking [173]. Increased levels of *pvmdr-1 *and *pvcrt*-o RNA in a single severe patient with vivax malaria however paved the way to the study of gene expression in association to resistance [174].

As suggested by the present data, 11 cases were reported in Brazilian travellers who live in the non-endemic area and occasionally go to the Amazon. Regarding the possibility of severe disease triggered by *P. vivax*, Travel Outpatient Clinics should emphasize to their clients the possible complications of this disease, still considered 'benign' in most of the educational folders and travellers' guides, especially because no good chemoprophylaxis against relapses related to this species is available to date. On top of that, retarded diagnosis and treatment outside the Amazon area contributes to the higher fatality rate of *P. falciparum *patients [18]. A similar situation could be observed for *P. vivax*, being this disease misdiagnosed as other febrile diseases.

Despite the increasing evidence of CQ-resistance worldwide, the Brazilian Ministry of Health still recommends CQ as the first line therapy for vivax treatment, considering that only one single study has properly shown ~10% of resistance in the area of Manaus [172]. The few available efficacy studies on ACT for the treatment of vivax were reviewed recently [175], and give good evidence for their use in vivax malaria, however, more studies are needed. Only recently the Brazilian Ministry of Health followed the WHO recommendations to manage vivax severe patients with parenteral artemisinin derivatives as if they had severe falciparum infection, considering that a submicroscopic mixed infection could be misdiagnosed in the routine TBS [106]. This recommendation was already stated by the famous Brazilian parasitologist Samuel Pessôa in his *Medical Parasitology *textbook, from 1967 [176]. Supportive therapy is even more neglected and there is virtually no study focusing in the clinical management of patients with severe vivax disease.

### Research priorities

**Figure 1:**
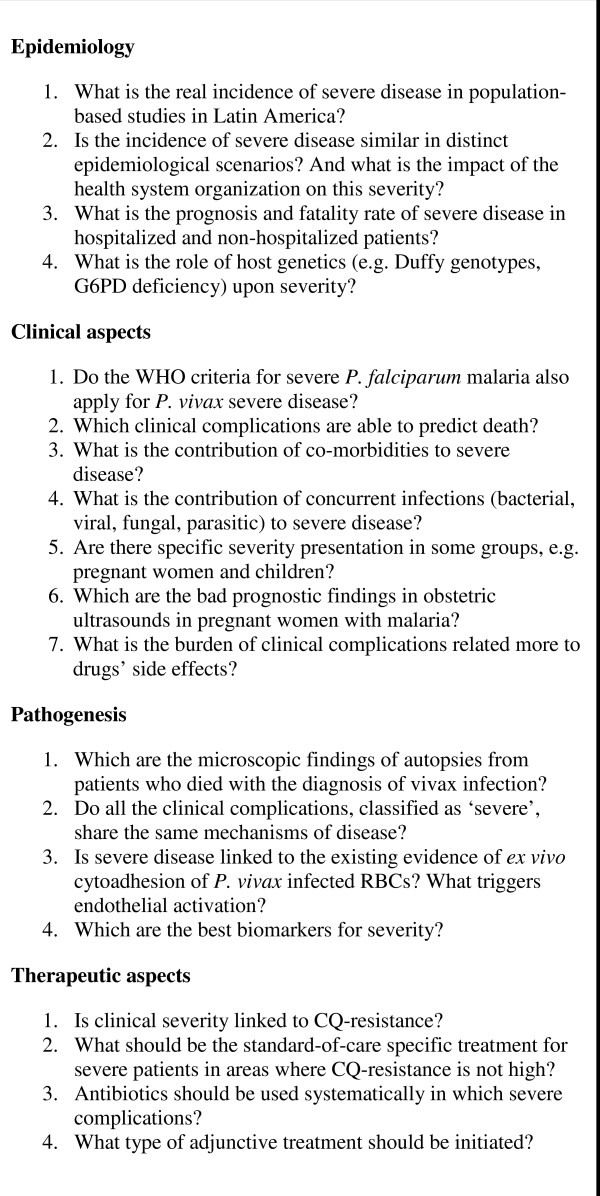
**Major research priorities in the study of severe vivax disease**.

There are actually many priorities in clinical research related to vivax disease. The major ones were discussed previously. Considering that asymptomatic infections due to *P. vivax *are even more common in endemic areas for both species [177], the likelihood of an asymptomatic patient becoming ill due to another microorganism is not improbable, which requests a good epidemiological characterization of the endemic area where the severe cases are being reported and systematic exclusion of mixed infections through PCR, due to the possibility of submicroscopic infection with *P. falciparum*. Another major priority in vivax research is the investigation of concurrent infections through systematic laboratory exclusion of the most prevalent infectious diseases in severe patients. In Figure 1, the major research questions are addressed.

## Conclusions

In the present systematic review, the major limitation was the fact that most of the information was retrieved from non-peer reviewed sources. However, it seems clear that vivax patients in Brazil are calling the attention of their physicians only recently. Like other infectious diseases, defining severity criteria is a major challenge. As an example, dengue fever specialists have defined 'warning signs' for dengue haemorrhagic fever, the most lethal complication of the infection due to dengue virus, which are early signs that should raise the suspicion of severe dengue but are not applied themselves to the final classification [178] as proper intervention can avoid the patient evolving to more severe stages. Sometimes in the literature potential 'warning signs' for severe vivax malaria are mistaken for severity criteria, which are those ultimately related to increased fatality. WHO severity criteria formerly developed for falciparum disease seem to apply reasonably to vivax disease as well, but there are clearly 'warning signs' that should motivate clinicians from the tropics to observe patients more closely, such as isolated thrombocytopaenia, isolated jaundice or the presence of chronic or acute co-morbidities. For example, during influenza outbreaks, the virus does not necessarily kill *per se*, but compromises the most vulnerable population and facilitates fatal secondary bacterial infections. The most common complications observed in the field are not necessarily the most frequently reported in the literature, sometimes biased by the uniqueness or exoticness of the cases reported. It is only after 1987 that these cases started to be reported in Brazil in indexed and non-indexed publications, which may simply parallel the increase in the absolute numbers of vivax cases in Brazil, culminating in the more frequent observation of rare clinical events triggered by this parasite. Publication bias may also impact the chronology of these complicated case reports, especially when research group leaderships based in the endemic areas start to look for clinical aspects more closely. It is noteworthy however that studies on pathogenesis must be careful when dealing with severe vivax disease as a single entity. The best approach is to study groups of patients with specific complications (e.g., severe anaemia or ARDS) in order to minimize the risk of heterogeneous groups with probable multifactor causality, including the diversity of host genetics. The amount of complications related to anti-malarial drug use is not negligible, especially primaquine. Multicentric studies using standard protocols, with the proper care of confirming mono-infection by more specific tools (e.g. PCR) and ruling out co-morbidities, are urgently needed to characterize the real spectrum of vivax disease worldwide. Tissues from deceased patients are also waited, in order to support more robust analyses of the mechanisms of death. Without that information, vaccine clinical trials against *P. vivax *will not be able to include among their endpoints the protection against the severe disease (essentially severe anaemia), which parallels the frequency of severe falciparum anaemia in some endemic areas. The recent discussion on malaria eradication will only succeed if the two parasites which most affect humans begin to be treated as distinct and not causing a single disease. Clinical characterization is the first step to estimate its burden and ultimately to plan any control strategy in the near future.

## Abbreviations

ACT: Artemisinin-Combination Therapy; AMA: Apical Membrane Antigen; API: Annual Parasite Incidence; ARDS: Acute Respiratory Distress Syndrome; CAPES: Coordination for the Improvement of Higher Education Personnel; CQ: Chloroquine; CRP: C-Reactive Protein; CSP: Circumsporozoite Protein; DIC: Disseminated Intravascular Coagulation; G6PD: Glucose-6-Phosphate-Dehydrogenase; HAV: Hepatitis A Virus; HBV: Hepatitis B Virus; HLEC: Human Lung Endothelial Cell; ICU: Intensive Care Unit; IL: Interleukin; INF: Interferon; ITP: Immune Thrombocytopenic Purpura; MODS: Multi-Organ Dysfunction Syndrome; MSP: Merozoite Surface Protein; PCR: Polymerase-Chain Reaction; PQ: Primaquine; RBC: Red Blood Cell; SCA: Sickle Cell Anaemia; SIVEP-Malaria: Epidemiological Surveillance Information System for Malaria; SOD: Superoxide dismutase; TBS: Thick Blood Smear; TGF: Tumor Growth factor; TNF: Tumor Necrosis Factor

## Competing interests

The authors declare that they have no competing interests.

## Authors' contributions

MVGL and MGCA conceived the review. MAAA performed the bibliographical research and retrieved the references. MVGL drafted the manuscript. MPGM, AMS, BMLM, FEME, FSSF, PB, AMRSV, MST, VSCDC, ARS and RSUS critically revised the document. All authors read and approved the final manuscript.

## References

[B1] Mendis K Sina BJ Marchesini P Carter R The neglected burden of *Plasmodium vivax *malaria Am J Trop Med Hyg 2001 64 97 106 10.4269/ajtmh.2001.64.97 11425182

[B2] Mendes C Dias F Figueiredo J Mora VG Cano J de Sousa B do Rosario VE Benito A Berzosa P Arez AP Duffy negative antigen is no longer a barrier to *Plasmodium vivax*-molecular evidences from the African West Coast (Angola and Equatorial Guinea) PLoS Negl Trop Dis 2011 5 e1192 10.1371/journal.pntd.0001192 PMC3119644 21713024

[B3] Guerra CA Howes RE Patil AP Gething PW Van Boeckel TP Temperley WH Kabaria CW Tatem AJ Manh BH Elyazar IR Baird JK Snow RW Hay SI The international limits and population at risk of *Plasmodium vivax *transmission in 2009 PLoS Negl Trop Dis 2010 4 e774 10.1371/journal.pntd.0000774 PMC2914753 20689816

[B4] Mueller I Galinski MR Baird JK Carlton JM Kochar DK Alonso PL del Portillo HA Key gaps in the knowledge of *Plasmodium vivax*, a neglected human malaria parasite Lancet Infect Dis 2009 9 555 566 10.1016/S1473-3099(09)70177-X 19695492

[B5] Price RN Tjitra E Guerra CA Yeung S White NJ Anstey NM Vivax malaria: neglected and not benign Am J Trop Med Hyg 2007 77 79 87 PMC2653940 18165478

[B6] Lacerda MVG Zackiewicz C Alecrim WD Alecrim MGC The neglected *Plasmodium vivax*: are researchers from endemic areas really concerned about new treatment options? Rev Soc Bras Med Trop 2007 40 489 490 10.1590/s0037-86822007000400026 17876480

[B7] Kochar DK Saxena V Singh N Kochar SK Kumar SV Das A *Plasmodium vivax *malaria Emerg Infect Dis 2005 11 132 134 10.3201/eid1101.040519 PMC3294370 15705338

[B8] Tjitra E Anstey NM Sugiarto P Warikar N Kenangalem E Karyana M Lampah DA Price RN Multidrug-resistant *Plasmodium vivax *associated with severe and fatal malaria: a prospective study in Papua, Indonesia PLoS Med 2008 5 e128 10.1371/journal.pmed.0050128 PMC2429950 18563962

[B9] Rogerson SJ Carter R Severe vivax malaria: newly recognised or rediscovered PLoS Med 2008 5 e136 10.1371/journal.pmed.0050136 PMC2429947 18563965

[B10] Reiter P From Shakespeare to Defoe: malaria in England in the Little Ice Age Emerg Infect Dis 2000 6 1 11 10.3201/eid0601.000101 PMC2627969 10653562

[B11] Austin SC Stolley PD Lasky T The history of malariotherapy for neurosyphilis. Modern parallels JAMA 1992 268 516 519 1619744

[B12] Picot S Is *Plasmodium vivax *still a paradigm for uncomplicated malaria? Med Mal Infect 2006 36 406 413 10.1016/j.medmal.2006.06.001 16842954

[B13] Galinski MR Barnwell JW *Plasmodium vivax: *who cares? Malar J 2008 7 Suppl 1 S9 10.1186/1475-2875-7-S1-S9 PMC2604873 19091043

[B14] Bassat Q Alonso PL Defying malaria: Fathoming severe *Plasmodium vivax *disease Nat Med 2011 17 48 49 10.1038/nm0111-48 21217683

[B15] WHO World Malaria Report 2010 2010

[B16] Saraiva MG Amorim RD Moura MA Martinez-Espinosa FE Barbosa MG Urban expansion and spatial distribution of malaria in the municipality of Manaus, State of Amazonas Rev Soc Bras Med Trop 2009 42 515 522 10.1590/s0037-86822009000500008 19967233

[B17] Gonçalves MJF Alecrim WD Non-planed urbanization as a contributing factor for malaria incidence in Manaus-Amazonas, Brazil Rev Salud Publica (Bogota) 2004 6 156 166 10.1590/s0124-00642004000200003 15382454

[B18] Oliveira-Ferreira J Lacerda MV Brasil P Ladislau JL Tauil PL Daniel-Ribeiro CT Malaria in Brazil: an overview Malar J 2010 9 115 10.1186/1475-2875-9-115 PMC2891813 20433744

[B19] Gama BE Lacerda MV Daniel-Ribeiro CT Ferreira-da-Cruz MF Chemoresistance of *Plasmodium falciparum *and *Plasmodium vivax *parasites in Brazil: consequences on disease morbidity and control Mem Inst Oswaldo Cruz 2011 106 Suppl 1 159 166 10.1590/s0074-02762011000900020 21881770

[B20] Genton B D'Acremont V Rare L Baea K Reeder JC Alpers MP Muller I *Plasmodium vivax *and mixed infections are associated with severe malaria in children: a prospective cohort study from Papua New Guinea PLoS Med 2008 5 e127 10.1371/journal.pmed.0050127 PMC2429951 18563961

[B21] Maitland K Williams TN Bennett S Newbold CI Peto TE Viji J Timothy R Clegg JB Weatherall DJ Bowden DK The interaction between *Plasmodium falciparum *and *P. vivax *in children on Espiritu Santo island, Vanuatu Trans R Soc Trop Med Hyg 1996 90 614 620 10.1016/s0035-9203(96)90406-x 9015495

[B22] Chagas C Hematological studies of impaludism MD Thesis 1903 Manguinhos Institute

[B23] Batista D The paludisme in the Amazon: a contribution to epidemiology, protozoology and clinics, study about the billiary-hemoglobinuric fever 1946 Rio de Janeiro: Imprensa Nacional

[B24] Santos-Ciminera PD Roberts DR Alecrim MG Costa MR Quinnan GV Jr Malaria diagnosis and hospitalization trends, Brazil Emerg Infect Dis 2007 13 1597 1600 10.3201/eid1310.070052 PMC2851511 18258018

[B25] Botelho C Guedes Barbosa LS Aquino JL Silva MD Meirelles SM Jardim JR Respiratory manifestations in *Plasmodium falciparum *and vivax malaria Rev Inst Med Trop Sao Paulo 1987 29 337 345 10.1590/s0036-46651987000600002 3331486

[B26] Moura EFA Kawachi J Mattos MNR Cavalcante RS Duarte AN Neonatal malaria: report of a case J Pediatr (Rio J) 1987 62 279 280

[B27] Severo V Severo MMS Behar PR Dossin TJ Laboratory profile of malarial patients J Bras Med 1994 67 141 154

[B28] Marques HH Vallada MG Sakane PT Boulos M Congenital malaria: case reports and brief review of literature J Pediatr (Rio J) 1996 72 103 105 10.2223/jped.590 14688962

[B29] Siqueira-Batista R Ramos AN Jr Pessanha BS Sforza-de-Almeida MP Potsch DF Chloroquine and cardiac arrhythmia: case report East Afr Med J 1998 75 117 119 9640837

[B30] Ventura AMRS Pinto AYN Silva RSU Calvosa VSP Silva Filho MG Souza JM *Plasmodium vivax *malaria in children and adolescents - epidemiological, clinical and laboratory features J Pediatr (Rio J) 1999 75 187 194 10.2223/jped.295 14685540

[B31] Pinheiro MCN Branco E Barata ACS Dantas RTS Fernandes WCS Malaria during the pregnancy and low birth weight from endemic areas in the Amazon Rev Para Med 2002 16 25 28

[B32] Jarude R Trindade R Tavares-Neto J Malaria in pregnant women of a public maternity of Rio Branco (Acre State, Brazil) Rev Bras Ginecol Obstet 2003 25 149 154

[B33] Lacerda MV Alexandre MA Santos PD Arcanjo AR Alecrim WD Alecrim MGC Idiopathic thrombocytopenic purpura due to vivax malaria in the Brazilian Amazon Acta Trop 2004 90 187 190 10.1016/j.actatropica.2003.12.001 15177145

[B34] Srinivas R Agarwal R Gupta D Severe sepsis due to severe falciparum malaria and leptospirosis co-infection treated with activated protein C Malar J 2007 6 42 10.1186/1475-2875-6-42 PMC1950478 17428347

[B35] Melo AM de Carvalho RA Figueiredo JF Vannucchi H Jordao A Jr Rodrigues ML Serum vitamin A levels in patients with ocular lesions attributable to non-complicated malaria in the Brazilian Amazon region Trans R Soc Trop Med Hyg 2004 98 485 488 10.1016/j.trstmh.2003.12.007 15186937

[B36] Braga MD Alcantara GC Silva CN Nascimento CG Cerebral malaria in Ceara: a case report Rev Soc Bras Med Trop 2004 37 53 55 10.1590/s0037-86822004000100014 15042185

[B37] Lomar AV Vidal JE Lomar FP Barbas CV Matos GJ Boulos M Acute respiratory distress syndrome due to vivax malaria: case report and literature review Braz J Infect Dis 2005 9 425 430 10.1590/s1413-86702005000500011 16410895

[B38] Vermehren R Cardoso AD Bulbol WS Franco M Coelho K Lorenzi F Nunes V Gonçalves J Report of a case of acute renal failure in *Plasmodium vivax *malaria J Bras Nefrol 2005 27

[B39] Curlin ME Barat LM Walsh DK Granger DL Noncardiogenic pulmonary edema during vivax malaria Clin Infect Dis 1999 28 1166 1167 10.1086/517767 10452658

[B40] Cabral PHO Andrade SD Alecrim WD Alecrim MGC Lacerda MVG Malaria and sickle cell anemia: report of complications and clinical management of three patients in a highly endemic area for *Plasmodium vivax *malaria in the Brazilian Amazon Case Rep Clin Pract Rev 2006 7 220 223

[B41] Lacerda MVG Oliveira SL Alecrim MGC Splenic hematoma in a patient with *Plasmodium vivax *malaria Rev Soc Bras Med Trop 2007 40 96 97 10.1590/s0037-86822007000100023 17486267

[B42] Santana MS Rocha MA Arcanjo AR Sardinha JF Alecrim WD Alecrim MGC Association of methemoglobinemia and glucose-6-phosphate dehydrogenase deficiency in malaria patients treated with primaquine Rev Soc Bras Med Trop 2007 40 533 536 10.1590/s0037-86822007000500008 17992408

[B43] Lacerda MV Hipolito JR Passos LN Chronic *Plasmodium vivax *infection in a patient with splenomegaly and severe thrombocytopenia Rev Soc Bras Med Trop 2008 41 522 523 10.1590/s0037-86822008000500021 19009202

[B44] Ramos WM Jr Sardinha JF Costa MR Santana MS Alecrim MG Lacerda MV Clinical aspects of hemolysis in patients with *P. vivax *malaria treated with primaquine, in the Brazilian Amazon Braz J Infect Dis 2010 14 410 412 10.1590/s1413-86702010000400017 20963329

[B45] Alexandre MA Ferreira CO Siqueira AM Magalhaes BL Mourao MPG Lacerda MVG Alecrim MGC Severe *Plasmodium vivax *Malaria, Brazilian Amazon Emerg Infect Dis 2010 16 1611 1614 10.3201/eid1610.100685 PMC3294402 20875292

[B46] Siqueira AM Alexandre MA Mourao MP Santos VS Nagahashi-Marie SK Alecrim MG Lacerda MV Severe rhabdomyolysis caused by *Plasmodium vivax *malaria in the Brazilian Amazon Am J Trop Med Hyg 2010 83 271 273 10.4269/ajtmh.2010.10-0027 PMC2911169 20682866

[B47] Melo GC Reyes-Lecca RC Vitor-Silva S Monteiro WM Martins M Benzecry SG Alecrim MG Lacerda MV Concurrent helminthic infection protects schoolchildren with *Plasmodium vivax *from anemia PLoS ONE 2010 5 e11206 10.1371/journal.pone.0011206 PMC2888569 20574512

[B48] Andrade BB Reis-Filho A Souza-Neto SM Clarencio J Camargo LM Barral A Barral-Netto M Severe *Plasmodium vivax *malaria exhibits marked inflammatory imbalance Malar J 2010 9 13 10.1186/1475-2875-9-13 PMC2837053 20070895

[B49] Chagas EC Nascimento CT Santana Filho FS Botto-Menezes CH Martinez-Espinosa FE Impact of malaria during pregnancy in the Amazon region Rev Panam Salud Publica 2009 26 203 208 10.1590/s1020-49892009000900003 20058829

[B50] Fragoso SC Alexandre MA Santos PJ Mourao MP Passos LN Magalhaes BM Siqueira AM Lacerda MV Hypovolaemic shock triggered by *P. vivax *infection in a patient with mild haemophilia A Haemophilia 2011 17 159 160 10.1111/j.1365-2516.2010.02350.x 20565547

[B51] Ferreira ME Gomes MD Vieira JL Methemoglobinemia in patients with *Plasmodium vivax *receiving oral therapy with primaquine Rev Soc Bras Med Trop 2011 44 113 115 10.1590/s0037-86822011000100026 21340422

[B52] Alecrim MGC Silva VMQ Melo MD Araújo JR Guerra MVF Silva NB Ferreira LCL Silva EB Alecrim WD Splenic rupture in malarial patients from the Instituto de Medicina Tropical de Manaus [abstract] Rev Soc Bras Med Trop 1995 28 151

[B53] Kalmar EMN Cassetari V Machado FR Alencar F Kirchgatter K Boulos M Tapajós R Severe malaria in patient with *Plasmodium vivax *infection: a case report [abstract] Rev Soc Bras Med Trop 1998 31 56

[B54] Sardinha YF Nunes SB Albuquerque SRL Marques HO Passos LNM Hemolytic anemia due to secondary cryoagglutinins and *P. vivax *malaria: report of two cases [abstract] Rev Soc Bras Med Trop 1998 31 144

[B55] Victoria MB Victoria F Coellho AHV Santos LO Alecrim MGC Thrombocytopenic purpura in patient with *Plasmodium vivax *malaria: case report [abstract] Rev Soc Bras Med Trop 1998 31 55

[B56] Zumpano JF Queiroz JN Rocha MOC Spontaneous splenic rupture in vivax malaria [abstract] Rev Soc Bras Med Trop 1998 31 46

[B57] Silva SL Santana Filho FS Arcanjo ARL Alecrim WD Alecrim MGC Clinical and hematological profile of hospitalized patients with vivax malaria and thrombocytopenia in the Fundação de Medicina Tropical do Amazonas, from January 2007 to September 1999 [abstract] Rev Soc Bras Med Trop 2000 33 348

[B58] Aragão PS Alecrim MGC Evangelista NMA Tavares AM Santana Filho FS Alecrim WD Clinical and laboratorial study on *Plasmodium vivax *malaria [abstract] Rev Soc Bras Med Trop 2001 34 341

[B59] Evangelista NMA Aragão DS Tavares AM Magalhães L Alexandre MAA Alecrim MGC Alecrim WD Neonatal malaria due to *Plasmodium vivax *in the Fundação de Medicina Tropical do Amazonas [abstract] Rev Soc Bras Med Trop 2002 35 344

[B60] Moura ACL Balbo BEP Cancela ALE Betti MH Heneine RAD Zumpano JF Death due to *P. vivax *in a non-endemic area [abstract] Rev Soc Bras Med Trop 2002 35 369

[B61] Park CHL Ferreira CB Bianchi CP Fazio FS Costa JC Padilha ARS Fonseca MO Boulos M Thrombocytopenia in patients with *Plasmodium vivax *malaria [abstract] Rev Soc Bras Med Trop 2002 35 370

[B62] Viana GMC Monteiro PS Nascimento MDSB Burarttini MN Severe malaria due to *P. vivax: *case report [abstract] Rev Soc Bras Med Trop 2002 35 367

[B63] Albuquerque BC Couto BB Moraes TC Couto S The relevance of clinical and laboratorial aspects in patients with *Plasmodium vivax *malaria hospitalized in the Fundação de Medicina Tropical do Amazonas, 2002 [abstract] Rev Soc Bras Med Trop 2003 36 280

[B64] Lacerda MVG Manso MRC Ferreira LCL Silva FM Santos PJT Alecrim WD Alecrim MGC Patient with vivax malaria and acute lung edema in the Brazilian Amazon: case report and literature review [abstract] Rev Soc Bras Med Trop 2003 36 281

[B65] Silva IBA Araújo JR Cleonardo A Ventura AM Pinto AY Linonati RMF Silva Filho M Valente MIA Castro JAA Souza JM Severe vivax malaria and non-cardiogenic pulmonary edema [abstract] Rev Soc Bras Med Trop 2003 36 273

[B66] Silva IBA Carneiro MS Araújo JR von Mühler CA Souza JM Vivax malaria and vasculitis: case evolving with death [abstract] Rev Soc Bras Med Trop 2004 37 275

[B67] Tavares AM Tavares AM Cavalcante CP Splenic subcapsullary hematoma in patient with *Plasmodium vivax *malaria [abstract] Rev Soc Bras Med Trop 2004 37 270

[B68] Penna J Zumpano JF Penna L Rocha MOC Case report: pulmonary manifestations of *Plasmodium vivax *malaria [abstract] Rev Soc Bras Med Trop 2005 38 379

[B69] Mello GS Mello Filho GB Rodrigues DCH Malarial coma due to *Plasmodium vivax *[abstract] Rev Soc Bras Med Trop 2006 39 106

[B70] Menezes CHAB Martinez-Espinosa FE Ferreira LCL Simplicio JL Frota AC Effects of malarial infection on the course of pregnancy and fetus in patients from the Fundação de Medicina Tropical do Amazonas [abstract] Rev Soc Bras Med Trop 2006 39 107

[B71] Oliveira RSM Santos VS Lacerda MVG Alecrim WD Hemolysis associated to acute renal failure in an immunocompetent patient with G6PD deficiency and vivax malaria: case report with favourable evolution [abstract] Rev Soc Bras Med Trop 2006 39 109

[B72] Bastos CJC Mascarenhas-Batista AV Freire M Amorim F Andrade M Gomes E Severe malaria due to *Plasmodium vivax*: case report [abstract] Rev Soc Bras Med Trop 2007 40 146

[B73] Campos LRP Mascheretti M Brasil RA Duarte MIS Evaluation of splenic immune response in patient infected with *Plasmodium vivax *[abstract] Rev Soc Bras Med Trop 2007 40 141

[B74] Gurgel RL Coutinho LI Su WCS Lacerda MVG Clinical manifestations of congenital vivax malaria in the Western Brazilian Amazon [abstract] Rev Soc Bras Med Trop 2007 40 147

[B75] Borzacov LMP Cardoso GA Siqueira GD Cardoso LAP Barbieri AB Fontes CJF Acute psychosis induced by chloroquine during vivax malaria treatment [abstract] Rev Soc Bras Med Trop 2008 41 221

[B76] Cardoso GA Siqueira GD Borzacov LMP Tristão FR Cardoso LAP Fontes CJF Leukemoid reaction caused by *Plasmodium vivax*: case report [abstract] Rev Soc Bras Med Trop 2008 41 221

[B77] Ohnishi MDO Ventura AM Linonati RMF Mendes MM Apolinário JHD Souza JM The lung in vivax malaria: case report [abstract] Rev Soc Bras Med Trop 2008 41 215

[B78] Menezes CHAB Silva SCL Farias LSR Bacelar BRB Bardaji A Meghna D Martinez-Espinosa FE Prevalence of anemia and *Plasmodium vivax *infection in routine antenatal attention in a primary care center in the City of Manaus [abstract] Rev Soc Bras Med Trop 2010 43 215

[B79] Ohnishi MDO Linonati RMF Silva AF Santos SEB Ventura AM Souza JM Pulmonary manifestations in patients infected with *Plasmodium vivax *and correlation with TNF-alfa and IL-12 polymorphisms [abstract] Rev Soc Bras Med Trop 2010 43 84

[B80] Urbaez-Brito JD Clinical and laboratorial characteristics of human malaria and hepatitis B virus association Master Dissertation 1995 University of Brasília, Tropical Medicine Department

[B81] Alecrim MGC Clinical aspects, resistance and parasitary polymorphism of *Plasmodium vivax *malaria in Manaus PhD Thesis 2000 University of Brasília, Tropical Medicine Department

[B82] Neves JJO Malaria in Pará: study of the clinical and laboratorial picture in infections caused by *Plasmodium vivax* Master Dissertation 2002 Federal University of Pará

[B83] Marques HO Hemostasis disturbances in patients with malaria Master Dissertation 2004 Federal University of São Paulo, Hematology Department

[B84] Oliveira MS Hematological characterization of children with vivax malaria diagnosed and treated in the Fundação de Medicina Tropical do Amazonas Master Dissertation 2004 University of the Amazonas State, Health Sciences School

[B85] Silva IBA Vivax malaria: clinical and laboratorial manifestations related to TNF-alpha PhD Thesis 2004 Federal University of Pará

[B86] Raposo CCBS Vivax malaria in Maranhão: epidemiological and clinical aspects Master Dissertation 2006 University of Brasília, Tropical Medicine Deprtment

[B87] Pereira MSS Study of malaria in pregnant and puerperal women in a public maternity in Manaus from 1999 to 2004 Master Dissertation 2006 Federal University of Amazonas, Health Sciences School

[B88] Guerreiro NSV Clinical and epidemiological study of *Plasmodium vivax *malaria in the State of Amapá Master Dissertation 2006 Federal University of Pará

[B89] Lacerda MVG Clinical manifestations and pathogenesis of malarial thrombocytopenia PhD Thesis 2007 University of Brasília, Tropical Medicine Department

[B90] Silva SBR Evaluation of frequency and factors associated to thrombocytopenia caused by *Plasmodium vivax* Master Dissertation 2009 Federal University of Mato Grosso

[B91] Fragoso SCP Study of 17 cases of autopsies from patients with the diagnosis of vivax malaria in a reference center in the Brazilian Amazon Master Dissertation 2010 University of the Amazonas State, Health Sciences School

[B92] Lança EFC Children under 14 years with malaria admitted to Intensive Care Units in the Brazilian Amazon: a case-control study with *Plasmodium vivax *patients Master Dissertation 2011 University of the Amazonas State, Health Sciences School

[B93] Karunaweera ND Wijesekera SK Wanasekera D Mendis KN Carter R The paroxysm of *Plasmodium vivax *malaria Trends Parasitol 2003 19 188 193 10.1016/s1471-4922(03)00036-9 12689650

[B94] Zimmerman PA Mehlotra RK Kasehagen LJ Kazura JW Why do we need to know more about mixed *Plasmodium *species infections in humans? Trends Parasitol 2004 20 440 447 10.1016/j.pt.2004.07.004 PMC3728821 15324735

[B95] Quintero JP Siqueira AM Tobon A Blair S Moreno A Arevalo-Herrera M Lacerda MV Valencia SH Malaria-related anaemia: a Latin American perspective Mem Inst Oswaldo Cruz 2011 106 Suppl 1 91 104 10.1590/s0074-02762011000900012 PMC4830680 21881762

[B96] Caicedo O Ramirez O Mourao MP Ziadec J Perez P Santos JB Quinones F Alecrim MG Arevalo-Herrera M Lacerda MV Herrera S Comparative hematologic analysis of uncomplicated malaria in uniquely different regions of unstable transmission in Brazil and Colombia Am J Trop Med Hyg 2009 80 146 151 19141853

[B97] Michon P Cole-Tobian JL Dabod E Schoepflin S Igu J Susapu M Tarongka N Zimmerman PA Reeder JC Beeson JG Schofield L King CL Mueller I The risk of malarial infections and disease in Papua New Guinean children Am J Trop Med Hyg 2007 76 997 1008 PMC3740942 17556601

[B98] Rodriguez-Morales AJ Sanchez E Vargas M Piccolo C Colina R Arria M Anemia and thrombocytopenia in children with *Plasmodium vivax *malaria J Trop Pediatr 2006 52 49 51 10.1093/tropej/fmi069 15980019

[B99] Poespoprodjo JR Fobia W Kenangalem E Lampah DA Hasanuddin A Warikar N Sugiarto P Tjitra E Anstey NM Price RN Vivax malaria: a major cause of morbidity in early infancy Clin Infect Dis 2009 10.1086/599041 PMC4337979 19438395

[B100] Katsuragawa TH Cunha RP Souza DC Gil LH Cruz RB Silva AA Tada MS Silva LHP Malaria and hematological aspects among residents to be impacted by reservoirs for the Santo Antonio and Jirau Hydroelectric Power Stations, Rondonia State, Brazil Cad Saude Publica 2009 25 1486 1492 10.1590/s0102-311x2009000700006 19578569

[B101] Ferreira MU Silva-Nunes M Bertolino CN Malafronte RS Muniz PT Cardoso MA Anemia and iron deficiency in school children, adolescents, and adults: a community-based study in rural Amazonia Am J Public Health 2007 97 237 239 10.2105/AJPH.2005.078121 PMC1781405 17194861

[B102] Cardoso MA Ferreira MU Camargo LM Szarfarc SC Anaemia, iron deficiency and malaria in a rural community in Brazilian Amazon Eur J Clin Nutr 1994 48 326 332 8055848

[B103] Wildig J Michon P Siba P Mellombo M Ura A Mueller I Cossart Y Parvovirus B19 infection contributes to severe anemia in young children in Papua New Guinea J Infect Dis 2006 194 146 153 10.1086/505082 16779719

[B104] Kochar DK Das A Kochar A Middha S Acharya J Tanwar GS Gupta A Pakalapati D Garg S Saxena V Subudhi AK Boopathi PA Sirohi P Kochar SK Thrombocytopenia in *Plasmodium falciparum, Plasmodium vivax *and mixed infection malaria: A study from Bikaner (Northwestern India) Platelets 2010 21 623 627 10.3109/09537104.2010.505308 21050055

[B105] Lacerda MV Mourao MP Coelho HC Santos JB Thrombocytopenia in malaria: who cares? Mem Inst Oswaldo Cruz 2011 106 Suppl 1 52 63 10.1590/s0074-02762011000900007 21881757

[B106] Guidelines for the treatment of malaria http://whqlibdoc.who.int/publications/2010/9789241547925_eng.pdf

[B107] Takaki K Aoki T Akeda H Kajiwara T Honda S Maeda Y Okada K Sawae Y A case of *Plasmodium vivax *malaria with findings of DIC (abstract) Kansenshogaku Zasshi 1991 65 488 492 10.11150/kansenshogakuzasshi1970.65.488 2071964

[B108] Lakhkar BB Babu S Shenoy V DIC in vivax malaria Indian Pediatr 1996 33 971 972 9141839

[B109] Mourao MP Lacerda MV Macedo VO Santos JB Thrombocytopenia in patients with dengue virus infection in the Brazilian Amazon Platelets 2007 18 605 612 10.1080/09537100701426604 18041652

[B110] Santana VS Lavezzo LC Mondini A Terzian AC Bronzoni RV Rossit AR Machado RL Rahal P Nogueira MC Nogueira ML Concurrent Dengue and malaria in the Amazon region Rev Soc Bras Med Trop 2010 43 508 511 10.1590/s0037-86822010000500007 21085859

[B111] Charrel RN Brouqui P Foucault C Lamballerie X Concurrent dengue and malaria Emerg Infect Dis 2005 11 1153 1154 10.3201/eid1107.041352 PMC3371802 16032797

[B112] WHO Severe falciparum malaria Trans R Soc Trop Med Hyg 2000 94 Supp 1 S1 S90 11103309

[B113] Illamperuma C Allen BL Pulmonary edema due to *Plasmodium vivax *malaria in an American Missionary Infection 2007 35 374 376 10.1007/s15010-007-6108-x 17721740

[B114] Pukrittayakamee S Chantra A Vanijanonta S White NJ Pulmonary oedema in vivax malaria Trans R Soc Trop Med Hyg 1998 92 421 422 10.1016/s0035-9203(98)91075-6 9850397

[B115] Tan LK Yacoub S Scott S Bhagani S Jacobs M Acute lung injury and other serious complications of *Plasmodium vivax *malaria Lancet Infect Dis 2008 8 449 454 10.1016/S1473-3099(08)70153-1 18582837

[B116] Anstey NM Russell B Yeo TW Price RN The pathophysiology of vivax malaria Trends Parasitol 2009 25 220 227 10.1016/j.pt.2009.02.003 19349210

[B117] Menendez C Romagosa C Ismail MR Carrilho C Saute F Osman N Machungo F Bardaji A Quinto L Mayor A Naniche D Dobano C Alonso PL Ordi J An autopsy study of maternal mortality in Mozambique: the contribution of infectious diseases PLoS Med 2008 5 e44 10.1371/journal.pmed.0050044 PMC2245982 18288887

[B118] Kochar SK Mahajan M Gupta RP Middha S Acharya J Kochar A Das A Kochar DK Acute attack of AIP (acute intermittent porphyria) with severe vivax malaria associated with convulsions: a case report J Vector Borne Dis 2009 46 307 309 19959859

[B119] Lampah DA Yeo TW Hardianto SO Tjitra E Kenangalem E Sugiarto P Price RN Anstey NM Coma associated with microscopy-diagnosed plasmodium vivax: a prospective study in Papua, Indonesia PLoS Negl Trop Dis 2011 5 e1032 10.1371/journal.pntd.0001032 PMC3110166 21666785

[B120] Cavasini MT Ribeiro WL Kawamoto F Ferreira MU How prevalent is *Plasmodium malariae *in Rondonia, western Brazilian Amazon? Rev Soc Bras Med Trop 2000 33 489 492 10.1590/s0037-86822000000500011 11064586

[B121] Costa AP Bressan CS Pedro RS Valls-de-Souza R Silva S Souza PR Guaraldo L Ferreira-da-Cruz MF Daniel-Ribeiro CT Brasil P Delayed diagnosis of malaria in a dengue endemic area in the Brazilian extra-Amazon: recent experience of a malaria surveillance unit in state of Rio de Janeiro Rev Soc Bras Med Trop 2010 43 571 574 10.1590/s0037-86822010000500020 21085872

[B122] Kochar DK Tanwar GS Khatri PC Kochar SK Sengar GS Gupta A Kochar A Middha S Acharya J Saxena V Pakalapati D Garg S Das A Clinical features of children hospitalized with malaria-a study from Bikaner, northwest India Am J Trop Med Hyg 2010 83 981 989 10.4269/ajtmh.2010.09-0633 PMC2963956 21036824

[B123] Kochar DK Das A Kochar SK Saxena V Sirohi P Garg S Kochar A Khatri MP Gupta V Severe *Plasmodium vivax *malaria: a report on serial cases from Bikaner in northwestern India Am J Trop Med Hyg 2009 80 194 198 19190212

[B124] Kochar DK Singh P Agarwal P Kochar SK Pokharna R Sareen PK Malarial hepatitis J Assoc Physicians India 2003 51 1069 1072 15260391

[B125] Khan FY El-Hiday AH Acute acalculous cholecystitis complicating an imported case of mixed malaria caused by *Plasmodium falciparum *and *Plasmodium vivax* Int J Infect Dis 2010 14 Suppl 3 e217 219 10.1016/j.ijid.2009.07.018 19932042

[B126] Kumar A Katiyar GP Mixed infection with *Plasmodium vivax *and *Salmonella typhi *in an infant Indian Pediatr 1995 32 243 244 8635791

[B127] Piyaphanee W Issarachaikul R Soontarach P Silachamroon U Concurrent salmonella bacteremia in P. vivax infection--a report of 2 cases at the Hospital for Tropical Diseases, Thailand Southeast Asian J Trop Med Public Health 2007 38 616 618 17882996

[B128] Fonseca JC Natural history of chronic hepatitis B Rev Soc Bras Med Trop 2007 40 672 677 10.1590/s0037-86822007000600015 18200423

[B129] Lacerda MV Mourao MP Santos PJ Alecrim MG Algid malaria: a syndromic diagnosis Rev Soc Bras Med Trop 2009 42 79 81 10.1590/s0037-86822009000100017 19287942

[B130] Stoppacher R Adams SP Malaria deaths in the United States: case report and review of deaths, 1979-1998 J Forensic Sci 2003 48 404 408 12665001

[B131] Bassat Q Guinovart C Sigauque B Mandomando I Aide P Sacarlal J Nhampossa T Bardaji A Morais L Machevo S Letang E Macete E Aponte JJ Roca A Menendez C Alonso PL Severe malaria and concomitant bacteraemia in children admitted to a rural Mozambican hospital Trop Med Int Health 2009 14 1011 1019 10.1111/j.1365-3156.2009.02326.x 19552643

[B132] Song JY Park CW Jo YM Kim JY Kim JH Yoon HJ Kim CH Lim CS Cheong HJ Kim WJ Two cases of *Plasmodium vivax *malaria with the clinical picture resembling toxic shock Am J Trop Med Hyg 2007 77 609 611 17978057

[B133] Anstey NM Handojo T Pain MC Kenangalem E Tjitra E Price RN Maguire GP Lung injury in vivax malaria: pathophysiological evidence for pulmonary vascular sequestration and posttreatment alveolar-capillary inflammation J Infect Dis 2007 195 589 596 10.1086/510756 PMC2532499 17230420

[B134] Carvalho BO Lopes SC Nogueira PA Orlandi PP Bargieri DY Blanco YC Mamoni R Leite JA Rodrigues MM Soares IS Oliveira TR Wunderlich G Lacerda MV Del Portillo HA Araujo MO Russell B Suwanarusk R Snounou G Renia L Costa FT On the Cytoadhesion of *Plasmodium vivax*-Infected Erythrocytes J Infect Dis 2010 202 638 647 10.1086/654815 20617923

[B135] Echeverri M Tobon A Alvarez G Carmona J Blair S Clinical and laboratory findings of *Plasmodium vivax *malaria in Colombia, 2001 Rev Inst Med Trop São Paulo 2003 45 29 34 10.1590/s0036-46652003000100006 12751319

[B136] Nosten F McGready R Simpson JA Thwai KL Balkan S Cho T Hkirijaroen L Looareesuwan S White NJ Effects of *Plasmodium vivax *malaria in pregnancy Lancet 1999 354 546 549 10.1016/s0140-6736(98)09247-2 10470698

[B137] Poespoprodjo JR Fobia W Kenangalem E Lampah DA Warikar N Seal A McGready R Sugiarto P Tjitra E Anstey NM Price RN Adverse pregnancy outcomes in an area where multidrug-resistant *Plasmodium vivax *and *Plasmodium falciparum *infections are endemic Clin Infect Dis 2008 46 1374 1381 10.1086/586743 PMC2875100 18419439

[B138] Martinez-Espinosa FE Daniel-Ribeiro CT Alecrim WD Malaria during pregnancy in a reference centre from the Brazilian Amazon: unexpected increase in the frequency of *Plasmodium falciparum *infections Mem Inst Oswaldo Cruz 2004 99 19 21 10.1590/s0074-02762004000100003 15057341

[B139] Poels PJ Dolmans WM Gabreels FJ Rhabdomyolysis associated with malaria tertiana in a patient with myoadenylate deaminase deficiency Trop Geogr Med 1993 45 83 86 8511818

[B140] Hamel CT Blum J Harder F Kocher T Nonoperative treatment of splenic rupture in malaria tropica: review of literature and case report Acta Trop 2002 82 1 5 10.1016/s0001-706x(02)00025-6 11904097

[B141] Lewallen S Harding SP Ajewole J Schulenburg WE Molyneux ME Marsh K Usen S White NJ Taylor TE A review of the spectrum of clinical ocular fundus findings in *P. falciparum *malaria in African children with a proposed classification and grading system Trans R Soc Trop Med Hyg 1999 93 619 622 10.1016/s0035-9203(99)90071-8 10717749

[B142] Lee JH Chin HS Chung MH Moon YS Retinal Hemorrhage in *Plasmodium vivax *Malaria Am J Trop Med Hyg 2010 82 219 222 10.4269/ajtmh.2010.09-0439 PMC2813160 20133995

[B143] Vitor-Silva S Reyes-Lecca RC Pinheiro TR Lacerda MV Malaria is associated with poor school performance in an endemic area of the Brazilian Amazon Malar J 2009 8 230 10.1186/1475-2875-8-230 PMC2768742 19835584

[B144] Williams TN Maitland K Phelps L Bennett S Peto TE Viji J Timothy R Clegg JB Weatherall DJ Bowden DK *Plasmodium vivax: *a cause of malnutrition in young children QJM 1997 90 751 757 10.1093/qjmed/90.12.751 9536339

[B145] Pereira PC Meira DA Curi PR Souza N Burini RC The malarial impact on the nutritional status of Amazonian adult subjects Rev Inst Med Trop Sao Paulo 1995 37 19 24 10.1590/s0036-46651995000100004 7569636

[B146] Ventura AM Pinto AY Silva RS Calvosa VS Silva Filho MG Souza JM *Plasmodium vivax *malaria in children and adolescents - epidemiological, clinical and laboratory features J Pediatr (Rio J) 1999 75 187 194 10.2223/jped.295 14685540

[B147] Silamut K White NJ Relation of the stage of parasite development in the peripheral blood to prognosis in severe falciparum malaria Trans R Soc Trop Med Hyg 1993 87 436 443 10.1016/0035-9203(93)90028-o 8249075

[B148] Andrade BB Reis-Filho A Souza-Neto SM Raffaele-Netto I Camargo LM Barral A Barral-Netto M Plasma superoxide dismutase-1 as a surrogate marker of vivax malaria severity PLoS Negl Trop Dis 2010 4 e650 10.1371/journal.pntd.0000650 PMC2850307 20386593

[B149] Santana MS Lacerda MVG Barbosa MGV Alecrim WD Alecrim MGC Glucose-6-phosphate dehydrogenase deficiency in an endemic area for malaria in Manaus: a cross-sectional survey in the Brazilian Amazon PLoS ONE 2009 4 e5259 10.1371/journal.pone.0005259 PMC2667256 19370159

[B150] Leslie T Briceno M Mayan I Mohammed N Klinkenberg E Sibley CH Whitty CJ Rowland M The impact of phenotypic and genotypic G6PD deficiency on risk of *Plasmodium vivax *infection: a case-control study amongst Afghan refugees in Pakistan PLoS Med 2010 7 e1000283 10.1371/journal.pmed.1000283 PMC2876136 20520804

[B151] Guindo A Fairhurst RM Doumbo OK Wellems TE Diallo DA X-linked G6PD deficiency protects hemizygous males but not heterozygous females against severe malaria PLoS Med 2007 4 e66 10.1371/journal.pmed.0040066 PMC1820604 17355169

[B152] Cavasini CE de Mattos LC Couto AA Couto VS Gollino Y Moretti LJ Bonini-Domingos CR Rossit AR Castilho L Machado RL Duffy blood group gene polymorphisms among malaria vivax patients in four areas of the Brazilian Amazon region Malar J 2007 6 167 10.1186/1475-2875-6-167 PMC2244634 18093292

[B153] Cavasini CE Mattos LC Couto AA Bonini-Domingos CR Valencia SH Neiras WC Alves RT Rossit AR Castilho L Machado RL *Plasmodium vivax *infection among Duffy antigen-negative individuals from the Brazilian Amazon region: an exception? Trans R Soc Trop Med Hyg 2007 101 1042 1044 10.1016/j.trstmh.2007.04.011 17604067

[B154] Albuquerque SR Cavalcante F de O Sanguino EC Tezza L Chacon F Castilho L dos Santos MC FY polymorphisms and vivax malaria in inhabitants of Amazonas State, Brazil Parasitol Res 2010 106 1049 1053 10.1007/s00436-010-1745-x 20162434

[B155] Costa FT Lopes SC Ferrer M Leite JA Martin-Jaular L Bernabeu M Nogueira PA Mourao MP Fernandez-Becerra C Lacerda MV Portillo HD On cytoadhesion of *Plasmodium vivax*: raison d'etre? Mem Inst Oswaldo Cruz 2011 106 Suppl 1 79 84 10.1590/s0074-02762011000900010 21881760

[B156] Campos FM Franklin BS Teixeira-Carvalho A Filho AL de Paula SC Fontes CJ Brito CF Carvalho LH Augmented plasma microparticles during acute *Plasmodium vivax *infection Malar J 2010 9 327 10.1186/1475-2875-9-327 PMC2998527 21080932

[B157] Andrade BB Araujo-Santos T Luz NF Khouri R Bozza MT Camargo LM Barral A Borges VM Barral-Netto M Heme impairs prostaglandin E2 and TGF-beta production by human mononuclear cells via Cu/Zn superoxide dismutase: insight into the pathogenesis of severe malaria J Immunol 2010 185 1196 1204 10.4049/jimmunol.0904179 20562262

[B158] Erel O Vural H Aksoy N Aslan G Ulukanligil M Oxidative stress of platelets and thrombocytopenia in patients with vivax malaria Clin Biochem 2001 34 341 344 10.1016/s0009-9120(01)00221-1 11440737

[B159] Araujo CF Lacerda MV Abdalla DS Lima ES The role of platelet and plasma markers of antioxidant status and oxidative stress in thrombocytopenia among patients with vivax malaria Mem Inst Oswaldo Cruz 2008 103 517 521 10.1590/s0074-02762008000600001 18949318

[B160] Grynberg P Fernandes Fontes CJ Martins Braga E Association between particular polymorphic residues on apical membrane antigen 1 (AMA-1) and platelet levels in patients with vivax malaria Clin Microbiol Infect 2007 10.1111/j.1469-0691.2007.01815.x 17727669

[B161] Fernandes AA Carvalho LJ Zanini GM Ventura AM Souza JM Cotias PM Silva-Filho IL Daniel-Ribeiro CT Similar cytokine responses and degrees of anemia in patients with *Plasmodium falciparum *and *Plasmodium vivax *infections in the Brazilian Amazon region Clin Vaccine Immunol 2008 15 650 658 10.1128/CVI.00475-07 PMC2292669 18256207

[B162] Wickramasinghe SN Abdalla SH Blood and bone marrow changes in malaria Baillieres Best Pract Res Clin Haematol 2000 13 277 299 10.1053/beha.1999.0072 10942626

[B163] Fernandez-Becerra C Yamamoto MM Vencio RZ Lacerda M Rosanas-Urgell A Del Portillo HA *Plasmodium vivax *and the importance of the subtelomeric multigene vir superfamily Trends Parasitol 2008 25 44 51 10.1016/j.pt.2008.09.012 19036639

[B164] Santos-Ciminera PD Alecrim MD Roberts DR Quinnan GV Jr Molecular epidemiology of *Plasmodium vivax *in the State of Amazonas, Brazil Acta Trop 2007 10.1016/j.actatropica.2007.02.013 17397788

[B165] Bussaratid V Walsh DS Wilairatana P Krudsood S Silachamroon U Looareesuwan S Frequency of pruritus in *Plasmodium vivax *malaria patients treated with chloroquine in Thailand Trop Doct 2000 30 211 214 10.1177/004947550003000410 11075653

[B166] Sahoo S Kumar M Sinha VK Chloroquine-induced recurrent psychosis Am J Ther 2007 14 406 407 10.1097/MJT.0b013e31802e4b0e 17667217

[B167] Katsuragawa TH Gil LHS Stabile RG Pires MG Bonini-Domingos CR Incidence evaluation of Glucose-6-Phosphate Dehydrogenase and hematological profile in Rondônia Rev Bras Hematol Hemoter 2004 26 268 273

[B168] Silva MC Santos EB Costal EG Filho MG Guerreiro JF Povoa MM Clinical and laboratorial alterations in *Plasmodium vivax *malaria patients and glucose-6-phosphate dehydrogenase deficiency treated with primaquine at 0.50 mg/kg/day Rev Soc Bras Med Trop 2004 37 215 217 10.1590/s0037-86822004000300004 15330059

[B169] Walsh DS Wilairatana P Tang DB Heppner DG Jr Brewer TG Krudsood S Silachamroon U Phumratanaprapin W Siriyanonda D Looareesuwan S Randomized trial of 3-dose regimens of tafenoquine (WR238605) versus low-dose primaquine for preventing *Plasmodium vivax *malaria relapse Clin Infect Dis 2004 39 1095 1103 10.1086/424508 15486831

[B170] Price RN Douglas NM Anstey NM New developments in *Plasmodium vivax *malaria: severe disease and the rise of chloroquine resistance Curr Opin Infect Dis 2009 22 430 435 10.1097/QCO.0b013e32832f14c1 19571748

[B171] Alecrim MG Alecrim W Macedo V *Plasmodium vivax *resistance to chloroquine (R2) and mefloquine (R3) in Brazilian Amazon region Rev Soc Bras Med Trop 1999 32 67 68 10.1590/s0037-86821999000100013 9927829

[B172] Santana Filho FS Arcanjo AR Chehuan YM Costa MR Martinez-Espinosa FE Vieira JL Barbosa MG Alecrim WD Alecrim MG Chloroquine-resistant *Plasmodium vivax*, Brazilian Amazon Emerg Infect Dis 2007 13 1125 1126 10.3201/eid1307.061386 PMC2878224 18214203

[B173] Orjuela-Sanchez P Santana Filho FS Machado-Lima A Chehuan YF Costa MR Alecrim MD Del Portillo HA Analysis of single-nucleotide polymorphisms in the crt-o and mdr1 genes of *Plasmodium vivax *among chloroquine resistant isolates from the Brazilian Amazon region Antimicrob Agents Chemother 2009 10.1128/AAC.00004-09 PMC2715622 19451296

[B174] Fernandez-Becerra C Pinazo MJ Gonzalez A Alonso PL Del Portillo HA Gascon J Increased expression levels of the pvcrt-o and pvmdr1 genes in a patient with severe *Plasmodium vivax *malaria Malar J 2009 8 55 10.1186/1475-2875-8-55 PMC2682795 19341456

[B175] Douglas NM Anstey NM Angus BJ Nosten F Price RN Artemisinin combination therapy for vivax malaria Lancet Infect Dis 2010 10 405 416 10.1016/S1473-3099(10)70079-7 PMC3350863 20510281

[B176] Pessoa SB Parasitologia Médica 1967 11 Rio de Janeiro: Guanabara-Koogan

[B177] Alves FP Durlacher RR Menezes MJ Krieger H Silva LH Camargo EP High prevalence of asymptomatic *Plasmodium vivax *and *Plasmodium falciparum *infections in native Amazonian populations Am J Trop Med Hyg 2002 66 641 648 10.4269/ajtmh.2002.66.641 12224567

[B178] Halstead SB Dengue Curr Opin Infect Dis 2002 15 471 476 10.1097/00001432-200210000-00003 12686878

